# Nutritional and Chemical-Physical Characterization of Fresh Pasta *Gnocchi* Prepared with Sea Water as New Active Ingredient

**DOI:** 10.3390/foods10112585

**Published:** 2021-10-26

**Authors:** Gabriella Santagata, Domenico Zannini, Salvatore Mallardo, Floriana Boscaino, Maria Grazia Volpe

**Affiliations:** 1 National Research Council, Institute of Polymers, Composites and Biomaterials, Via Campi Flegrei 34, 80078 Pozzuoli, Italy; gabriella.santagata@ipcb.cnr.it (G.S.); domenico.zannini@ipcb.cnr.it (D.Z.); salvatore.mallardo@ipcb.cnr.it (S.M.); 2 Institute of Food Science, National Research Council, Via Roma 64, 83100 Avellino, Italy; floriana.boscaino@isa.cnr.it

**Keywords:** *Gnocchi*, sea water, micro-macro elements, volatile profile, thermal properties, morphological analysis

## Abstract

This study shows the chemical–physical and nutritional results obtained using food-grade sea water for the preparation of fresh pasta *Gnocchi* with respect to those prepared with tap water. *Gnocchi* obtained by mixing the flour with seawater (GSW) were compared with traditional *Gnocchi* made with tap water (GTW). The contents of sodium chloride, macro and micro elements, volatile molecules profile, thermal properties, and morphological analysis were investigated in both *Gnocchi* types. The analysis of chlorides showed that the samples prepared with sea water had a significantly lower NaCl content after cooking in comparison with those prepared with tap water. These results were also confirmed by the inductively coupled plasma (ICP) analysis for sodium content. The profiles of the volatile molecules acquired by gas chromatography-mass spectrometry (GC-MS) evidenced significant differences between the groups of aromatic molecules of the two typologies of samples. Morphological analysis evidenced that both raw and cooked GSW *Gnocchi* were structurally tightened whereas GTW *Gnocchi* showed a labile and weak macromolecular network. In addition, GSW *Gnocchi* was more thermally stable than GTW *Gnocchi*, as evidenced by thermogravimetric analysis (TGA).

## 1. Introduction

*Gnocchi* is a typically Italian culinary preparation, mainly used as a first course. In general, unless otherwise specified, the term “*Gnocchi*” identifies “those of potatoes”. As with many other Italian specialties, there are as many variations of gnocchi as there are regions in Italy and Italian housewives. *Gnocchi* dates back to Roman times, and at that time it did not contain potatoes but was made of a porridge-like dough. The potato variety only came after the potato had taken its way to Europe [1].

Anyway, even though both are high in carbohydrates, regular pasta may have less effect on blood sugar levels. Nevertheless, it is important to note that this study compared plain potatoes to pasta—not *Gnocchi*, which are prepared by kneading potato, wheat, and eggs. *Gnocchi* contains a good share of protein and cooks very quickly in boiling water (2 to 3 min). Served with various sauces (gorgonzola, butter and sage, tomato and parmesan, amongst the most famous), they became a very popular dish, even overseas, where *Gnocchi* are known almost as ‘’pasta’’ when speaking of a classical Italian “primo piatto” [2]. Besides being tasty, gnocchi-type pastas are relatively easy to prepare and consume. However, like pastas, *Gnocchi* are also commonly cooked in salt-water and, after their recovery from water, they preserve about 10% in weight of salt. Several studies have highlighted a causal relationship between salt intake and blood pressure [3]. Randomized trials demonstrate that a lower salt intake is associated with a reduced risk of cardiovascular disease, all-cause mortality, and other conditions, such as kidney disease, stomach cancer, and osteoporosis [4,5].

For the above reasons and in order to prepare a novel healthy and functional *Gnocchi* pasta type, keeping up the good natural flavor and taste, sea water has been substituted to tap water and used as an active ingredient of *Gnocchi* dough. The main characteristic of sea water is its ionic salt content [6]. Salinity indicates the quantity of salts dissolved in marine waters coming from the constant supply of substances from over land flowing rivers, submarine volcanoes, and decomposing marine organisms. Specifically, salinity measures the quantity of salt present in a kilogram of sea water; for ocean waters, it is around 35% [7]. The use of sea water as a new active ingredient of *Gnocchi* formulation can enhance the chemical-nutritional properties of this kind of pasta by increasing the micro and macro elements and decreasing or completely eliminating the content of added salt since cooking is performed in unsalted boiling water; moreover, GSW higher thermal stability, the mineral content is preserved during and after gnocchi cooking.

Thus, the aim of this research was the formulation of *Gnocchi* using edible sea water in place of tap water and investigate the properties of fresh pasta (*Gnocchi*) prepared with edible sea water (GSW) and compare them with the ones of *Gnocchi* prepared with tap water (GTW). Specifically, the percentage content of sodium chloride, concentration of macro and micro elements content together with the volatile molecules profile were evaluated, compared, and discussed. In addition, the morphological analysis (SEM) of raw and cooked GSW and GTW cryogenically delaminated fractures was performed and the study of their thermal stability was assessed by thermogravimetric analysis (TGA).

## 2. Materials and Methods

*Gnocchi* samples were supplied by Pastificio Artigianale Leonessa, Via Don Minzoni 231, 80040 Cercola, Napoli, Italy. Sea water, produced from Abba Blu srl (San Sperate CA, free of microorganisms and free of substances harmful for health, suitable for human consumption, in compliance with current legislation Italy), was kindly supplied from Asset srl Portici (NA), Italy. *Gnocchi* dough consisted of wheat flour 00, potatoes and tap water (identification code GTW) or food-grade sea water (identification code GSW).

### 2.1. Gnocchi Cooking Process and Water Absorption

In order to evaluate the properties of *Gnocchi*, specimens of GSW and GTW were dropped in unsalted boiling water for three minutes, gently recovered with a strained, allocated in Petri dishes, and left to dry at room temperature under a ventilated hood. In addition, GTW were also cooked in 10% *w/v* of salted water in order to reproduce the usual cooking methodology. The water absorption capacity of *Gnocchi* samples was determined after drying the samples in an oven until constant weight and the result was expressed as percentage water absorption.

Calculation formula:

%AW = W_after cooking_ − W_before cooking_/W_before cooking_ × 100 (1)

where AW = Absorption water after cooking, W_after cooking_ = *Gnocchi* weight after cooking, W_before cooking_ = *Gnocchi* weight before cooking.

### 2.2. Determination of the Sodium Chloride Content (the Mohr Method)

Approximately 2 g of the raw and cooked GSW and GTW samples were kept in an oven at 105 °C until their constant weight was reached; then, the samples were soaked in 40 mL of water-ultratrace. Then, the solutions were stirred for 2 h at room temperature, centrifugated for 10 min at 4000 rpm, filtered in 50 mL volumetric flask, and made up to volume with water-ultratrace. The pH of the solution was adjusted with 0.1 N sodium hydroxide up to a value of 8.0. About 2 mL of K_2_CrO_4_ indicator was added to 20 mL of solution that was titrated with 0.1 N silver nitrate until endpoint. In parallel, a blank test was carried out by titrating 20 mL of distilled water with 0.1 N silver nitrate until endpoint in the presence of 2 mL of K_2_CrO_4_ indicator.

By knowing the stoichiometry and moles consumed at the end point, the amount of chloride was determined. From the milligrams of Cl^−^ ion, the mg of NaCl of the sample is obtained.

### 2.3. Mineralization of Samples and Determination of Micro-Macro Elements: Inductively Coupled Plasma-Optical Emission Spectrometers Analysis ICP-OES

*Gnocchi* samples underwent acid digestion according to the method of Volpe et al. [8], slightly modified. Five milliliters of HNO_3_ and 2.5 mL of H_2_O_2_ were added to 0.5 g of dried sample. The mixture was digested at 240 °C until the solution became transparent, (Heating digester DK VELP Scientifica). After cooling, the solution was diluted with water-ultratrace up to a final volume of 25 mL in a volumetric flask and microfiltrated. The clear and plain solution was analyzed with (ICP-OES). The elemental analysis of macro, micro, and trace elements was performed by ICP-OES with iCAP 7000 Series (Thermo Scientific, Waltham, MA, USA), equipped with ASX-520 autosampler (CETAC™). A calibration curve was constructed using a standard mix solution containing all analyzed elements. The final concentration of the elements was expressed as mg element kg^−1^ dry weight.

### 2.4. Determination of Volatile Compounds by Solid Phase Microextraction Gas Chromatography/Mass Spectrometer Analysis (SPME-GC/MS)

Volatile compounds of both raw and cooked GTW and GSW samples were determined by solid-phase micro-extraction (SPME) coupled to gas-chromatography/mass spectrometry (GC/MS), according to the modified method of Di Renzo et al. [9]. GTW and GSW samples were cut into pieces of 2–3 mm. For each SPME analysis, 3 g of sample was placed in a 20 mL headspace vial. The sample was allowed to equilibrate at 40 °C for 5 min at 250 rpm using the automatic sampling system Gerstel MPS2 (Gerstel GmbH & Co., Mülheim, Germany). The analysis was conducted by a GC/MS system (Agilent 7890/5975 Inert, Agilent, Santa Clara, CA, USA) equipped with the above-mentioned autosampler with helium as carrier gas (1.5 mL/min). A coated Divinylbenzene/carboxen/Polydimethylsiloxane (DVB/CAR/PDMS) fiber (Sigma-Aldrich S.r.l., Milan, Italy) was exposed to the headspace of the sample for 30 min maintaining the sample at 40 °C. The fiber was desorbed for 10 min at 240 °C in the injection unit, in splitless mode. The separation was carried out in a capillary column HP Innowax (Agilent Technologies, Santa Clara, CA, USA) (30 m × 0.25 mm id. × 0.50 μm film thickness). The GC oven temperature program started at 40 °C for 3 min, then was ramped to 240 °C at 5 °C/min and kept the final temperature for 10 min. The mass spectrometer operated with an ion source at 230 °C, quadrupole temperature of 150 °C, 70 eV electron energy, acquiring in TIC mode from m/z 33 to 300 uma. Identification of volatile compounds was achieved by comparing mass spectra with the Wiley library (Wiley7, NIST 05). The proportion of each compound was estimated by dividing its mean area by the total area of the chromatogram and expressed as a percentage. Blank experiments were conducted in two different modalities: blank of the fiber and blank of the empty vial. All the analyses were performed in duplicate.

### 2.5. Thermogravimentric Analysis (TGA)

Thermogravimetric analyses (TGA) were carried out with a Mettler Thermogravimetric Analyzer Mod. TG. Measurements were performed on samples of about 4–6 mg, placed in open ceramic crucibles, and heated from room temperature to 600 °C at 20 °C/min in a nitrogen atmosphere, with a nominal gas flow rate of 30 mL/min. Before the tests, a blank curve was measured and subtracted from the single thermograms, to correct from instrumental drift [10].

### 2.6. Scanning Electron Microscopy (SEM)

Morphological analysis of both gnocchi after baking in tap and sea water and dried post-cooking residual fractions were carried out on cryogenically fractured surfaces by scanning electron microscopy (SEM) (Quanta 200 FEG, FEI, Eindhoven, The Netherlands) in high vacuum mode, using a secondary electron detector and an accelerating voltage of 20.0 kV. Before the electron microscopy observation, the fractured surfaces were coated with Au–Pd alloy with an SEM coating device (MED 020, Bal-Tec AG, Tucson, AZ, USA). The coating provided the entire sample surfaces with a homogeneous layer of metal of 18 ± 0.2 nm.

### 2.7. Statistical Analysis

The mean and standard deviation were calculated for each experimental parameter. Differences among the sample bread were determined for each volatile compound by analysis of variance (ANOVA), and the results were considered significant if the associated *p* values were below 0.05 (Tanagra 1.4 software).

## 3. Results

### 3.1. Water Absorption

After cooking, *Gnocchi* samples prepared with tap and sea water exhibited low water absorption. This outcome was expected considering the drastic structural changing undergone by starch upon heating and following cooling. In fact, it is well known that chemically, starch is mostly composed of linear amylose and highly branched amylopectin, providing a native crystalline material [11,12]. When heated in water, starch granules undergo spontaneous destructurization; they become hydrated, swell, and are transformed into a paste. The granule structure collapses due to the melting of crystallites, unwinding of double helices, and breaking of hydrogen bonds. These changes are collectively referred to as starch gelatinization. On cooling, the disaggregated starch chains retrograde gradually into partially ordered structures of crystallites that differ from those in native granules [13]. Moreover, the starch molecules’ re-crystallization increases over the cooling time. As a result, a labyrinth structure that blocks the water molecules’ absorption occurs, because of the hydrophobic behavior of the crystalline zones that, backwards, are more prone to expel water from the crystalline zones. This outcome, widely accepted in the literature, can withstand the very low water absorption recorded for both Gnocchi samples [14].

Anyway, higher water absorption values were found for GTW samples (+0.8%) as compared with GSW (+0.1%). This can be related to the presence of micro and macro elements in sea water able to strengthen the already crystalline network formed upon cooling involving both carbohydrate and protein polar residues of the *Gnocchi* matrix [15]. As a consequence, the available water binding sites were strongly reduced and substituted by the ionic physical binding of salts and polar residues of macromolecular structures, as shown by Razzaq et al., in their similar investigation [16]. This hypothesis will be confirmed by the results of the thermal analysis, as following discussed.

### 3.2. Evaluation of Sodium Chloride Content in Raw/Cooked GTW and GSW Samples

The weight percentage of sodium chloride was evaluated on raw and cooked samples and the results are reported in Table 1. Firstly, it deserves highlighting that *Gnocchi* prepared with tap water and cooked in 10% *w/v* of salted water absorbed about 65% of NaCl in their edible network. Actually, chefs and pasta producers recommend using 10 g of salt per 100 g of pasta in 1 L of cooking water, since it is well known that salt enhances the food taste. If these indications are respected, the salt weight gain is certainly remarkable; usually, gnocchi prepared with tap water in the dough and cooked in salted water can absorb up to 3% by weight of NaCl [17]. Anyway, according to the World Health Organization (WHO), the consumption of sodium chloride must be decreased during the day to about a total of 4 g per individual, a hardly reached limit if considering that most hidden salt is ingested with packaged foods [18].

In order to decrease the intake of salt, it is advisable to strongly limit its use during home cooking; therefore the possibility of obtaining healthy dishes prepared with less amount of sodium chloride without renouncing to the excellent sensorial properties of mouthwatering delicacies could represent a beneficial output for consumers and an interesting scientific approach finalized to obtain increasingly healthy foods [19]. In addition, it is important to emphasize that the right flavor depends on several factors, such as the type of salt used, the type of pasta, and obviously the sensitivity of the consumers’ taste buds [20]. Finalized to prepare palatable and healthy *Gnocchi* pasta type, sea water was used as an ingredient of dough and the following *Gnocchi* were cooked in unsalted tap water.

From the analysis of Table 1, it is worth highlighting that when *Gnocchi* are prepared in unsalted water, they partially release the starting salt content as there is a tendency to achieve a saline balance between the water and the *Gnocchi*, as expected. Thus, when salt is added to the water, the pasta must absorb it during the rehydration process and cooking, in a homogeneous and quantitatively correct way. If salt is not added to water, the internal salt of the *Gnocchi* will diffuse in the cooking water until salt absorption/desorption equilibrium is reached [17]. In fact, while GTW samples lost 72.4% of starting dough salt, GSW specimens released only 30% of NaCl. This outcome could be ascribed to the strong physical network developed between the polar groups of starch and protein-based components of *Gnocchi* (flour and potatoes) and the salts naturally available in sea water [21]. Indeed, it is likely that starch and protein carboxyl, amide, hydroxyl residues can develop ionic interactions with salt cations and hydrogen bonding, thus providing a tightened polymer-based structure responsible for the physical entrapment of NaCl in GSW samples [22]. It is not the case that these samples evidenced higher physical and thermal stability, demonstrated by both SEM and TGA analysis. Besides the above chemico-physical properties, *Gnocchi* prepared with sea water evidenced a greater content of beneficial macroelements, as discussed in the following section.

### 3.3. Evaluation of Macro and Micro Elements of GTW and GSW Samples by ICP-OES Method

In Table 2, GSW and GTW macro and micro element contents are detailed. All the measurements were performed in triplicate and the deviation standard for all the samples ranged between 0.01–0.08%. It is worth underscoring that, except for sodium ions, all the macro-micro element concentrations were considerably higher in GSW samples; this outcome could be due to their significant presence in sea water physical binding with polar residues of both carbohydrate and protein residues inside *Gnocchi* dough. In fact, macro-micro elements could physically interact with potatoes amylopectin/amylose polar residues, as following demonstrated by morphological and thermal properties and widely reported in the literature [23].

Potassium, calcium, and magnesium contribute to developing vital cell functions, particularly as heart excitability is concerned. They are key elements for myocardial movement and activation of enzymatic systems [24,25].

Calcium and magnesium results are higher than those reported by Nalepa et al. [26]. Magnesium is contained in many foods; however, about 80% of this metal is lost during food processing and as a consequence, a large percentage of people all over the world do not meet the minimum daily magnesium requirement [27]. This mineral activates several biochemical reactions all necessary for proper functioning of the human body. Magnesium has a role in controlling the transport of most minerals, including calcium, potassium, and sodium, in order to exploit their function such as nerve conduction, muscle contraction, and in maintaining heart rhythm [28].

Potassium is an essential mineral and also an electrolyte, mainly found in the intracellular fluid where it is the most important positive ionic strength and is required for normal cell function because of its role in maintaining intracellular fluid volume and transmembrane electrochemical gradients. Along with sodium, it regulates the right heartbeat to tune and stabilize the blood pressure, tunes the metabolism of carbohydrates and proteins, stimulates the elimination of toxic residues in the body [29]. Zinc and copper are essential elements for the human body and they result in chronic and acute effects in case of their deficiency [30]. About 99% of Zinc is intracellular while the rest is in plasma. The function of zinc in cells and tissues is dependent on metallic proteinase and these enzymes are associated with reproductive, neurological, immune, dermatological systems. It is essential for normal spermatogenesis and maturation, genomic integrity of sperm, for normal organogenesis, proper functioning of neurotransmitters, proper development of the thymus, proper epithelialization in wound healing, taste sensation, secretion of pancreatic and gastric enzymes [31]. Copper plays a very important role in human metabolism particularly because it allows many critical enzymes to function properly. In human blood, copper is principally distributed between the erythrocytes and in the plasma. In erythrocytes, 60% of copper occurs as copper-zinc metallic-enzyme superoxide dismutase [32].

The iron concentrations were lower than those reported by Al-Mussali and Al-Gahri [33], while zinc concentrations resulted higher. Finally, as expected, the Iodine content was significantly higher in GSW samples. Iodine is an important micronutrient element and is required for the synthesis of thyroid hormones regulating several important physiological processes. In fact, an iodine-deficient diet causes a wide spectrum of illnesses, including goiter and mental retardation. The possibility of iodine oral intake with *Gnocchi* could represent a valid method to enrich the diet of this element [34,35]. These data suggested that *Gnocchi* prepared with food sea water (GSW) could represent a healthy and nutrient functional food able to increase the uptake of essential macro-micro nutrients.

Finally, Cd and Pb concentrations were minor of 0.005 μg kg^−1^ for all samples, showing no environmental contamination [36,37].

### 3.4. Evaluation of Volatile Compounds by HS-SPME-GC/MS Analysis

Forty-four volatile organic compounds (VOCs) belonging to various chemical classes, were identified through SPME-GC/MS and reported in Table 3. The main compounds belonged to eight classes, aldehydes (13), ketones (10), alcohols (7), furans (3), acids (10), terpenes (4), esters (1), and phenols (2). Notwithstanding there are few available studies concerning the organoleptic quality of gnocchi and in general fresh pasta and, in particular, very poor data on flavor characterization. Some studies suggest that VOCs pasta might be affected by raw ingredients flavors (e.g., flour, potatoes, water) and by the different processing phases, such as blending, forming, packaging, and storage [38,39,40]. On the other hand, the VOCs of *Gnocchi* pasta might derive from sugar microbial metabolism that enhances by the addition of water during mixing and dough formation phases, as (alcohols, 2,3-butanedione, 3-hydroxy-2-butanone, esters, and acids), and by enzymatic oxidation or autoxidation of fatty raw materials (e.g., aliphatic aldehydes, ketones, acids and alkylfuran). The aldehydes were the most represented volatile molecules in the headspace of gnocchi samples and increased during cooking. In detail, the compound hexanal was the most abundant aldehyde in all gnocchi samples investigated; while aldehydes and ketones increase during cooking; instead, alcohols, furans, and acids decrease with cooking. During the cooking process, the starch-lipid, probably included as complexes with amylose may be released, becoming susceptible to thermal oxidation, and although the enzymes (e.g., lipoxygenase and hydroperoxidase lyase) involved in the production of aldehydes are heat denatured, the high temperatures accelerate autoxidation, a process that does not require enzymatic catalysis [38]. In accordance with some authors, in general, increases in aldehydes and ketones during handling and cooking processes are associated with a decrease in the content of alcohol and ester compounds. The loss of alcohol compounds can be due to the high solubility of alcohols in water an effect greatly enhanced by the cooking process because of the large volume of water employed [40,41]. The terpenes compounds might derive from raw ingredients. There is no evidence in the literature on the influence of water origin (SW and TW) on the production of volatile compounds in gnocchi samples.

### 3.5. Thermogravimetric Analysis (TGA, DTG)

**Figure 1:**
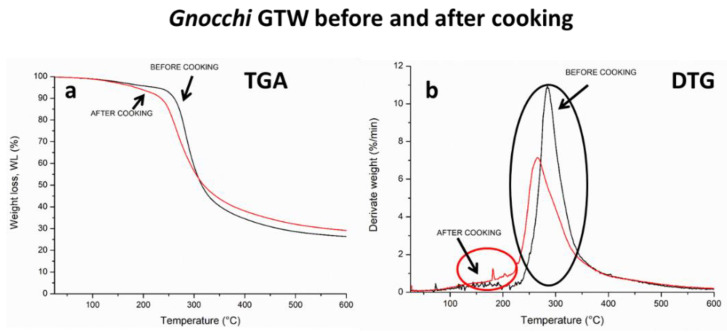
(**a**) TGA thermograms and (**b**) DTG curves of *Gnocchi* GTW before and after cooking.

In Figure 1a,b, TGA and derivative (DTG) thermograms of GTW samples before and after cooking are reported. All the curves were normalized with respect to starting sample weights.

From the analysis of GTW thermograms, it is possible to observe a lower water content after cooking (black curve). In fact, in *Gnocchi* dough, both starch and proteins are involved in physical interactions due to the presence of polar residues in both macromolecular structures; thus, starch and protein can form a gel when hydrated and heated and the gelling strength is influenced by several factors such as heating rate, holding time and temperature, pH, ionic strength, minerals contents, all factors resulting in a complex physicochemical environment [42]. After starch–protein gelation, upon *Gnocchi* heating and cooling, a chemico-physical aggregation (hydrogen bonds, hydrophobic interaction, and Van der Waals forces) of the polymers occur, likely due to starch retrogradation, a process of recrystallization that takes place during food cooling and storage. In particular, after amylose releasing in boiling water, the branched amylopectin fraction, organizes in a tight and compact structure drastically reducing the available water binding sites. For this reason, as expected, the water content in cooked and cooled gnocchi is reduced (Figure 1a). The presence of amino acids influences starch pasting and retrogradation inducing a tight and strong physical network [43]. The main differences are correlated to the loss of food product quality, such as gnocchi staling, loss of viscosity, and of course, syneresis, as evidenced by Mohamed et al. [44]. Moreover, from the DTG curves (Figure 1b), it is possible to observe two thermal degradation phenomena: the first one is related to the water losing kinetics (red ring) at about 50–100 °C and the second one is due to starch–protein thermal degradation rate at around 300–350 °C [13]. No substantial differences can be found in the decomposition rate of the starch–protein macromolecular network of raw and cooked *Gnocchi.*

**Figure 2:**
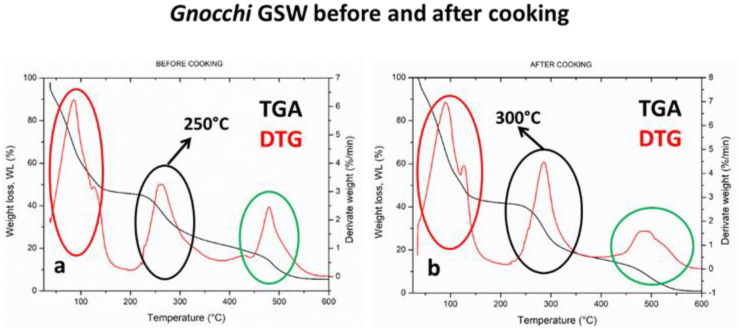
(**a**) TGA thermograms and (**b**) DTG curves of *Gnocchi* GSW before and after cooking.

As far as GSW samples are concerned, different thermal profiles are reported for better highlighting their substantial differences before and after cooking. Indeed, both TGA and DTG thermograms are overlapped for raw (Figure 2a) and cooked (Figure 2b) GSW samples. Firstly, in both cases, three thermal degradation phenomena occurred: the first one was ascribed to water releasing in the range of 50–100 °C [45]; the second thermal step was associated with starch–protein decomposition (black ring); the third was correlated to salt decomposition at about 450–500 °C (green curve). In fact, from DTG curves, the maximum degradation rate of raw GSW starch–protein fraction occurred at 250 °C, whereas the same phenomenon was shifted towards a substantially higher temperature (300 °C) for cooked GSW samples. Furthermore, a sharper single peak can be observed for cooked GSW samples; this outcome was in some way expected considering that the ionic and hydrogen bonding developed between sea water salts and polysaccharide and protein polar residues of potatoes and flour, resulted in a more tightened polymer network, highlighting a single pattern of degradation kinetics [46]. The higher stability of the polymer network in the cooked sample was due to the presence of the minerals enhancing the structural tightening. Thus, it is very probable that the micronutrients naturally occurring in sea water are preserved in GSW samples also after cooking, as confirmed by the micro/macro-nutrient analysis detailed in Table 2 and by the presence of salts thermal peak at around 500 °C, far above the degradation kinetics of the polymer network. This outcome was confirmed by morphological analysis, as discussed in the following section.

### 3.6. Scanning Electron Microscopy (SEM)

**Figure 3:**
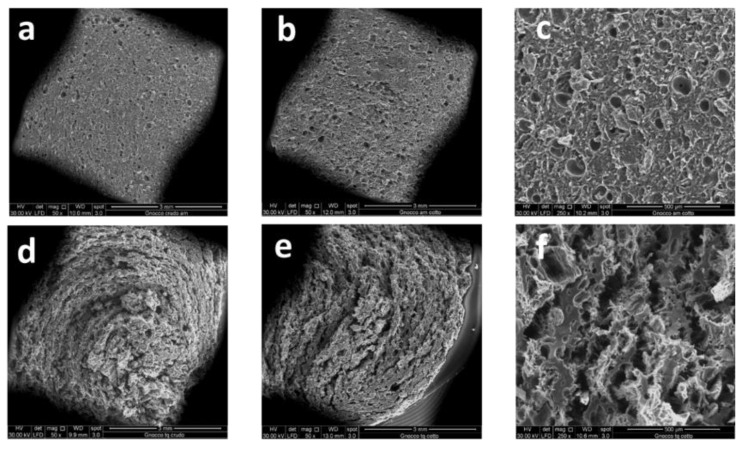
SEM micrographs of *Gnocchi* fractured surfaces: raw (**a**) and cooked in saline water (**b**,**c**) and raw (**d**) and cooked in tap water (**e**,**f**).

The SEM micrographs of fractured surfaces of raw and cooked *Gnocchi* prepared with tap and sea water are reported in Figure 3. *Gnocchi* samples were cooked in unsalted water, in order to respect the right balance of dispersed inner ions and to correctly evaluate the morphological structure of GSW and GTW fractured surfaces. The micrographs related to raw (Figure 3a) and cooked (Figure 3b,c) GSW samples evidenced a tight structure of interconnected polymer regions homogeneously distributed. In fact, strong physical interactions occurred between the polar residues of starch, protein, and the ions naturally present in sea water, resulting in an optimal adhesion and stickiness of all the components, substantially different from the weak and labile network of samples cooked in salty water. It can be concluded that the sea water, used as a new ingredient of gnocchi dough improved their textural characteristics. Moreover, it is amazing to highlight that no substantial structural differences were observed before and after *Gnocchi* cooking, thus meaning that the physical binding resulted in a three-dimensional stable network in which mostly sea water macro-micro nutrients were preserved also after cooking at high temperature. This outcome both confirms the effectiveness of sea water as a protective agent of *Gnocchi* morphology, appearance and texture after the thermal treatment, and supports the previous findings of GSW higher thermal stability. Nevertheless, some small circular voids were observed both in raw and in cooked GSW samples, as evidenced in Figure 3c. They were likely due to the formation of some air bubbles during *Gnocchi* kneading and preserved also after cooking, further confirming the strength of the GSW polymer network. As far as GTW samples are concerned, in Figure 3d–f, the micrographs of raw (Figure 3d) and cooked (Figure 3e,f) samples are reported. A more heterogeneous and weaker network with the presence of irregular voids dispersed throughout the inner delaminated surface could be highlighted [47]. In particular, from the higher magnification of cooked GTW samples of Figure 3f, discrete domains of higher material agglomeration were found, typical of a less tight and uneven polymer network [48]. In fact, during cooking, starch gelatinization and protein coagulation, occurring nearly at the same temperature, causes major structural changes resulting in textural hardness and stickiness loss.

## 4. Conclusions

This study evidenced that *Gnocchi*, one of the most loved and appreciated Italian dishes, kneaded with sea water represents a valuable option to obtain a novel functional food in which the enhanced nutritional and healthy micro-macro nutrients together with the higher profile of volatile molecules, confer high added value to the traditional *Gnocchi* prepared with tap water, providing a beneficial output for consumers and an interesting scientific approach finalized to obtain increasingly healthy foods.

The strongly decreasing sodium content, absolutely recommended from food guidelines for a healthy diet to reduce the risk of serious health diseases, represents a challenging and terrific strong point in the use of the active ingredient. Subsequent evaluations with a trained panel will also highlight the sensorial aspects of gnocchi kneaded with sea water in order to evaluate the influence of the novel ingredient and cooking process on the tasty and palatable acceptability of the finished product, highly important for the food and gastronomy sciences community.

## Figures and Tables

**Table 1:** NaCl content of raw and cooked GTW and GSW *Gnocchi*.

Sample	% NaCl Content	% NaCl Gain (+), Losing (−)
Raw GTW	1.81	-
GTW cooked in 10% *w/v* salted water	3.00	+65.7
GTW cooked in unsaltedWater	0.50	−72.4
Raw GSW	2.00	-
GSW cooked in unsaltedWater	1.40	−30.0

**Table 2:** Macro-micro elements of GTW and GSW samples expressed in mg/kg.

Sample/Elements	Na	Ca	K	Mg	Fe	Cu	Sr	Zn	I
GTW	80	240	1870	285	11.735	2.179	1.970	11.130	0.010
GSW	50	340	2500	700	17.690	2.905	4.607	14.095	0.041

**Table 3:** Volatile organic compounds (VOCs) identified by SPME-GC/MS in *Gnocchi* samples.

Sample/Elements	Raw GTW	Raw GSW	GTW Cooked in Unsalted Water	GSW Cooked in Unsalted Water
Aldehydes				
2-methylbutanal	0.54 ± 0.02	1.09 ± 0.02	1.17 ± 0.02	0.21 ± 0.01
3-methylbutanal	1.10 ± 0.07	2.11 ± 0.28	1.71 ± 0.02	1.96 ± 0.08
Pentanal	2.69 ± 0.14	Nd	2.93 ± 0.38	1.55 ± 0.06
Hexanal Octanal	56.31 ± 0.31	14.28 ± 0.03	66.36 ± 0.50	39.88 ± 1.53
2-heptenal	0.31 ± 0.01	1.12 ± 0.00	Nd	3.64 ± 0.16
Nonanal	Nd	Nd	Nd	3.34 ± 0.08
2-octenal	2.33 ± 0.04	5.11 ± 0.15	3.51 ± 0.09	6.51 ± 0.18
decanal	Nd	Nd	Nd	0.94 ± 0.0
Benzaldehyde	Nd	Nd	Nd	2.13 ± 0.01
Tot	1.98 ± 0.07	5.07 ± 0.09	3.51 ± 0.02	5.87 ± 0.28
Ketones				
2-Propanone	3.52 ± 0.07	2.63 ± 0.22	2.23 ± 0.09	5.85 ± 0.42
2,3-butanedione	Nd	1.33 ± 0.01	Nd	Nd
2-heptanone	Nd	2.45 ± 0.10	Nd	3.02 ± 0.03
1-hepten-3-one	Nd	Nd	0.47 ± 0.02	Nd
Acetoin	Nd	1.69 ± 0.00	0.84 ± 0.02	2.05 ± 0.08
2,3-octanedione	0.17 ± 0.00	Nd	0.34 ± 0.03	Nd
6-methyl-5-hepten-2-one	0.22 ± 0.00	0.49 ± 0.02	0.27 ± 0.04	0.77 ± 0.04
3,5-octadien-2-one	0.61 ± 0.01	2.04 ± 0.10	1.07 ± 0.03	2.54 ± 0.08
3-octen-2-one	Nd	Nd	0.55 ± 0.02	Nd
Tot	4.52 ± 0.07	10.62 ± 0.23	5.77 ± 0.09	14.23 ± 0.63
Alcohols				
ethanol	0.18 ± 0.01	0.17 ± 0.00	Nd	Nd
1-Pentanol	3.92 ± 0.13	4.68 ± 0.14	3.05 ± 0.18	4.00 ± 0.15
1-Hexanol	3.56 ± 0.00	6.42 ± 0.14	2.43 ± 0.01	5.77 ± 0.15
3,5-octadien-2-ol	0.36 ± 0.01	Nd	0.23 ± 0.01	Nd
1-octen-3-ol	1.20 ± 0.05	1.73 ± 0.02	1.36 ± 0.10	1.49 ± 0.03
Benzenemethanol	Nd	0.58 ± 0.01	Nd	Nd
Tot	9.22 ± 0.15	13.58 ± 0.26	7.07 ± 0.06	11.26 ± 0.02
Furans				
2-methylfuran	0.52 ± 0.00	4.90 ± 0.08	Nd	Nd
2-ethylfuran	1.49 ± 0.02	3.93 ± 0.14	1.33 ± 0.05	2.09 ± 0.02
2-pentylfuran	4.70 ± 0.21	8.73 ± 0.42	4.91 ± 0.12	3.34 ± 0.02
Tot	6.70 ± 0.22	17.57 ± 0.20	6.24 ± 0.16	5.43 ± 0.04
Acids				
Acetic acid	3.58 ± 0.11	5.61 ± 0.13	Nd	Nd
2-methylbutanoic acid	0.24 ± 0.01	Nd	Nd	Nd
pentanoic acid	Nd	0.48 ± 0.03	Nd	Nd
Hexanoic acid	1.21 ± 0.07	1.88 ± 0.01	0.82 ± 0.01	Nd
Heptanoic acid	0.41 ± 0.02	0.78 ± 0.02	0.24 ± 0.01	Nd
Octanoic acid	0.53 ± 0.01	1.60 ± 0.01	Nd	Nd
Nonanoic acid	0.53 ± 0.02	2.41 ± 0.09	Nd	Nd
Dodecanoic acid	0.49 ± 0.02	Nd	Nd	Nd
Tetradecanoic acid	1.32 ± 0.09	Nd	0.21 ± 0.00	Nd
Hexadecanoic acid	3.21 ± 0.13	4.55 ± 0.06	0.22 ± 0.00	Nd
Tot	11.51 ± 0.23	17.31 ± 0.09	1.49 ± 0.00	Nd
Terpenes				
alpha pinene	0.86 ± 0.00	1.42 ± 0.04	Nd	1.04 ± 0.03
delta 3-carene	1.55 ± 0.08	3.35 ± 0.14	Nd	Nd
p-cymene	Nd	0.68 ± 0.02	Nd	Nd
tot	2.41 ± 0.08	5.45 ± 0.08	Nd	1.04 ± 0.02
Esters				
Ethyl Acetate	Nd	5.81 ± 0.04	Nd	Nd
Tot	Nd	5.81 ± 0.04	Nd	Nd
Phenols				
3-ethylphenol	0.31 ± 0.01	0.61 ± 0.01	Nd	Nd
Phenol	0.08 ± 0.00	0.28 ± 0.02	0.25 ± 0.02	Nd
Tot	0.38 ± 0.01	0.89 ± 0.07	0.25 ± 0.02	Nd
Terpenes				
alpha pinene	0.86 ± 0.00	1.42 ± 0.04	Nd	1.04 ± 0.03
delta 3-carene	1.55 ± 0.08	3.35 ± 0.14	Nd	Nd
p-cymene	Nd	0.68 ± 0.02	Nd	Nd
Tot	0.38 ± 0.01	0.89 ± 0.07	0.25 ± 0.02	Nd

Results are reported as A% = Area Peak Compound/Area Peak Total Compounds (A% ± SD), Nd (not detected).

## Data Availability

Not Applicable.
